# Case Report of a Fatal Antifreeze Ingestion with a Record High Level and Impressive Renal Crystal Deposition

**DOI:** 10.1155/2016/3101476

**Published:** 2016-09-22

**Authors:** Heidi L. Erickson

**Affiliations:** Department of Pulmonary, Critical Care, and Occupational Medicine, University of Iowa Hospitals and Clinics, 200 Hawkins Drive, Iowa City, IA 52242, USA

## Abstract

Ethylene glycol, methanol, and diethylene glycol are readily available in many household and commercially available products. While these alcohols are relatively nontoxic themselves, their acidic metabolites are toxic and can result in significant morbidity and mortality. Herein we report a lethal case of massive ethylene glycol ingestion in a suicide with a record high level (1254 mg/dL) and images of the histologic examination of the kidneys revealing impressive calcium oxalate crystal deposition. Autopsy findings also showed evidence of mild cerebral edema.

## 1. Introduction

Ethylene glycol, methanol, and diethylene glycol are readily available in many household and commercially available products such as antifreeze, windshield washer fluid, and fuel additives. Ethylene glycol is colorless and odorless and has a sweet taste; these properties render it a source for accidental and intentional ingestion. Unlike ethanol and isopropyl alcohol whose toxicity is related to its alcohol moiety, the toxicity of ethylene glycol, methanol, and diethylene glycol is related to their acidic metabolites resulting from oxidations by alcohol dehydrogenase (ADH) and aldehyde dehydrogenase [1]. Glycolic acid and oxalic acid are the toxic metabolites of ethylene glycol. The 2014 US National Poison Data System suggests that, of the substances most frequently involved in human ingestion, the frequency of exposures to the toxic alcohols remains prevalent [2].

The cardinal features of ethylene glycol toxicity are inebriation or encephalopathy, a wide anion gap metabolic acidosis, calcium oxalate crystal deposition in the urine, and acute kidney injury. Nearly all organ systems can be affected leading to multisystem organ failure [3]; calcium oxalate crystals can be found in the brain, lungs, kidneys, and heart. Clinically evident toxicity is usually seen with ingestion of 1 g/kg (approximately 2.6 oz in a 75 kg person) or serum ethylene glycol levels of >20 mg/dL [3].

## 2. Case Presentation

A 37-year-old man with a history of depression was found unresponsive at his home with two empty one-liter bottles of antifreeze and a suicide note nearby. Emergency Medical Services were contacted and the patient was taken to a local community hospital. On arrival to the outside hospital, he had a Glasgow Coma Score of 4 with Kussmaul respirations and normal hemodynamics; he was endotracheally intubated. Initial arterial blood gas showed a pH of 6.79 with a pCO_2_ of 37 mmHg, pO_2_ of 115 mmHg, and bicarbonate of 5.5 mEq/L. Screening tests for salicylates, acetaminophen, and drugs of abuse were negative. His blood chemistries showed a blood glucose of 232 mg/dL, bicarbonate of 8.4 mEq/L, creatinine of 1.5 mg/dL, and anion gap of 33. He was transferred to our facility and the poison control center was contacted and alerted to suspected ethylene glycol ingestion.

Upon arrival to our facility, approximately 3.5 hours after initial presentation, the patient was unresponsive on no sedation. His blood pressure was trending down, though still within normal limits, and he was tachycardic and afebrile. His pupils were sluggish but reactive, and he had spontaneous respirations. His laboratory values were notable for a venous pH of 6.78, anion gap of 43, plasma osmolality of 828 mOsm/kg, osmolality gap of 483 mOsm/kg, and a serum lactate of >30 mEq/L. Calcium oxalate crystals were detected in his urine. His ethylene glycol level was 1254 mg/dL. Screening for other volatile alcohols was negative.

**Figure 1:**
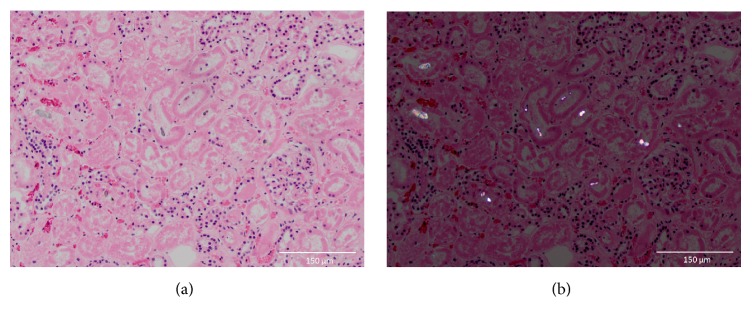
Histologic examination of the kidney revealing calcium oxalate crystals which appear birefringent under polarized light (b).

The patient received a loading dose of fomepizole on arrival and was started on intermittent hemodialysis. Within minutes after the initiation of dialysis, he suffered a cardiac arrest. Following return of spontaneous circulation, he was started on pressor support and converted to continuous renal replacement therapy. He remained on fomepizole and was also treated with thiamine and folate. Over the course of several hours, the patient's pupils became fixed and dilated, and he developed progressive hypotension, apnea, autonomic dysfunction, and flattening of the waves on his electroencephalogram. The patient died 22 hours after initial presentation. Autopsy revealed mild cerebral edema with hypoxic-ischemic neuronal injury and calcium oxalate crystals in the renal tubules (Figure 1).

## 3. Discussion

In situations where the presenting circumstances are unknown, a severe metabolic acidosis with a wide anion gap and osmolar gap can be the first clue to ethylene glycol poisoning; the presence of urinary calcium oxalate crystals is essentially diagnostic. Early diagnosis and treatment of such toxicity are paramount to successful outcomes. This case is remarkable for the amount of ethylene glycol consumed and an initial serum level of 1254 mg/dL, which is nearly 1.5 times higher than previously reported record high levels [3]. Unfortunately, despite knowledge of the ingestion at the time of presentation and treatment with evidence based therapies, the patient did not survive.

The mainstays of treatment for ethylene glycol poisoning include inhibition of ADH to prevent the formation of the toxic acidic metabolites and hemodialysis [4]. ADH inhibition can be achieved with the administration of ethanol, a competitive ADH substrate, or fomepizole, a potent competitive ADH inhibitor [4]. Ethanol has unpredictable pharmacokinetics necessitating intensive monitoring of serum concentrations, can worsen the encephalopathy, and causes hypoglycemia. Moreover, ethanol therapy is to be combined with hemodialysis [1]. Fomepizole, however, has well characterized pharmacokinetics, does not require monitoring of serum concentrations, and can obviate the need for hemodialysis in many cases [1]. Adjunct thiamine and pyridoxine can also be given to promote metabolism of the toxic metabolites to benign end products [3]. The American Academy of Clinical Toxicology practice guidelines state that fomepizole should be used as the first-line agent and ethanol should be used if fomepizole is not available [5].

In summary, we describe a lethal case of ethylene glycol poisoning with a record high level and impressive renal tubule calcium oxalate deposition. It is important to have a high index of suspicion for toxic alcohol poisoning and to start treatment early if ingestion is suspected or in the presence of a metabolic acidemia of unknown etiology. Collaboration with a poison control center or toxicologist is advised.

## References

[1] McMartin K., Jacobsen D., Hovda K. E. (2016). Antidotes for poisoning by alcohols that form toxic metabolites. *British Journal of Clinical Pharmacology*.

[2] Mowry J. B., Spyker D. A., Brooks D. E., Mcmillan N., Schauben J. L. (2015). 2014 annual report of the American association of poison control centers National Poison Data System (NPDS): 32nd Annual Report. *Clinical Toxicology*.

[3] Davis D. P., Bramwell K. J., Hamilton R. S., Williams S. R. (1997). Ethylene glycol poisoning: case report of a record-high level and a review. *Journal of Emergency Medicine*.

[4] Brent J., McMartin K., Phillips S. (1999). Fomepizole for the treatment of ethylene glycol poisoning. *The New England Journal of Medicine*.

[5] Barceloux D. G., Krenzelok E. P., Olson K., Watson W., Miller H. (1999). American academy of clinical toxicology practice guidelines on the treatment of ethylene glycol poisoning. *Journal of Toxicology—Clinical Toxicology*.

